# A stem cell based *in vitro* model of NAFLD enables the analysis of patient specific individual metabolic adaptations in response to a high fat diet and AdipoRon interference

**DOI:** 10.1242/bio.054189

**Published:** 2021-01-25

**Authors:** Nina Graffmann, Audrey Ncube, Soraia Martins, Aurelian Robert Fiszl, Philipp Reuther, Martina Bohndorf, Wasco Wruck, Mathias Beller, Constantin Czekelius, James Adjaye

**Affiliations:** 1 Institute for Stem Cell Research and Regenerative Medicine, Heinrich Heine University Düsseldorf, Medical faculty, Moorenstrasse 5, 40225 Düsseldorf, Germany; 2 Institute of Organic Chemistry and Macromolecular Chemistry, Heinrich-Heine University Düsseldorf 40225, Düsseldorf, Germany; 3 Institute for Mathematical Modeling of Biological Systems, Heinrich-Heine University Düsseldorf, Düsseldorf, Germany; 4 Systems Biology of Lipid Metabolism, Heinrich-Heine University Düsseldorf 40225, Düsseldorf, Germany

**Keywords:** NAFLD, AdipoRon, FGF21, Metabolism, Hepatocyte differentiation, Hepatocyte-like cells

## Abstract

Non-alcoholic fatty liver disease (NAFLD) is a multifactorial disease. Its development and progression depend on genetically predisposed susceptibility of the patient towards several ‘hits’ that induce fat storage first and later inflammation and fibrosis. Here, we differentiated induced pluripotent stem cells (iPSCs) derived from four distinct donors with varying disease stages into hepatocyte like cells (HLCs) and determined fat storage as well as metabolic adaptations after stimulations with oleic acid. We could recapitulate the complex networks that control lipid and glucose metabolism and we identified distinct gene expression profiles related to the steatosis phenotype of the donor. In an attempt to reverse the steatotic phenotype, cells were treated with the small molecule AdipoRon, a synthetic analogue of adiponectin. Although the responses varied between cells lines, they suggest a general influence of AdipoRon on metabolism, transport, immune system, cell stress and signalling.

## Abstract (precis)

**Summary:** A stem cell based *in vitro* model of NAFLD recapitulates regulatory networks and suggests a steatosis associated phenotype. AdipoRon treatment influences metabolism, immune system, cell stress and signalling.

## INTRODUCTION

Non-alcoholic fatty liver disease (NAFLD) or steatosis is the hepatic manifestation of the metabolic syndrome and affects up to 35% of the general population in the western hemisphere, with increasing tendencies (Cohen et al., 2011). It is a multifactorial disease with sedentary lifestyle, an imbalance in calorie uptake and energy expenditure, obesity, diabetes, insulin resistance, and also genetic predisposition playing crucial roles in its development. However, so far it is poorly understood how these factors interact and why people react very differently to similar dietary conditions.

When the liver encounters a surplus of calories that is not matched by appropriate energy expenditure, it starts storing triacylglycerides in lipid droplets (LDs). This first stage is still reversible but the accumulation of LDs in hepatocytes represents the first of several ‘hits’ that eventually impair hepatocyte function. Further hits, e.g. by inflammation or oxidative stress can lead to non-alcoholic steatohepatitis (NASH) in 30% of patients (Cohen et al., 2011). From there the disease can proceed to cirrhosis and hepatocellular carcinoma, which finally requires liver transplantation (Wong et al., 2015).

Although storage of fat in relatively inert LDs prevents lipotoxicity (Neuschwander-Tetri, 2017), it takes up a lot of space and resources in hepatocytes, thus diminishing their ability to adapt the metabolism to the bodies energy needs.

Hepatic metabolism is controlled by a complex network of signalling pathways that integrate information on nutrient availability and energy needs within the liver and peripheral organs (Bechmann et al., 2012). One of the signalling molecules that influences hepatic metabolism is adiponectin. It is an adipokine – a cytokine synthesized by adipocytes. Adiponectin levels are inversely correlated with bodyweight as well as with insulin sensitivity (Vuppalanchi et al., 2005; Wruck et al., 2015; Kadowaki and Yamauchi, 2005). It signals via two distinct receptors, adiponectin receptor (ADIPOR) 1 and 2. ADIPOR1 is ubiquitously expressed, while ADIPOR2 is predominantly present in the liver (Yamauchi et al., 2003; Felder et al., 2010). AdipoR signalling activates the key metabolic regulators 5’ adenosine monophosphate-activated protein kinase (AMPK) (predominantly via AdipoR1) and peroxisome proliferator-activated receptor (PPAR)α (predominantly via AdipoR2) (Yamauchi et al., 2007), which in turn are responsible for co-ordinating key metabolic pathways (Liu et al., 2012). In hepatocytes, adiponectin reduces gluconeogenesis and lipogenesis (Combs and Marliss, 2014). In adipocytes and skeletal muscle, it increases insulin-mediated glucose uptake and utilisation while it also stimulates insulin secretion by pancreatic beta cells in response to glucose stimulation (Ruan and Dong, 2016). Importantly, adiponectin is also capable of reducing whole body inflammation levels, mainly by stimulating M2 macrophage proliferation and activity and reducing M1 macrophage activities (Luo and Liu, 2016). However, several studies have also described a pro-inflammatory role of adiponectin, especially in the context of rheumatoid arthritis (Koskinen et al., 2011; Ehling et al., 2006).

In 2013, a small molecule with adiponectin-like function, which activates both receptors, was discovered and named AdipoRon (Okada-Iwabu et al., 2013). AdipoRon improves insulin sensitivity and reduces fasting blood glucose levels in high fat diet-induced obese mice. On a high fat diet, it reduced liver triacylglyceride levels in wild-type (wt) mice and prolonged the lifespan of *db/db* mice (Okada-Iwabu et al., 2013).

To date, most studies on NAFLD have been performed in rodents which have marked metabolic differences compared to humans (Santhekadur et al., 2018). We recently established a human *in vitro* model of NAFLD based on induced pluripotent stem cell (iPSC) derived hepatocyte like cells (HLCs) (Graffmann et al., 2016). This model allows us to (i) analyse the development of NAFLD taking into account different disease-associated genotypes that might explain the different courses of disease development, and (ii) to study the effect of potential treatments that should prevent or revert the NAFLD phenotype.

Here, we differentiated four iPSCs lines derived from donors with distinct grades of steatosis into HLCs and studied their responses to fatty acid overload and AdipoRon treatment. While all cell lines efficiently exhibited hallmarks of steatosis, the exact molecular responses to the treatment were highly variable, which can be attributed, at least in part, to variations in the individual genetic background of the donors.

## RESULTS

### HLCs can be derived from iPSCs of donors with distinct grades of NAFLD

In order to validate our previously published *in vitro* model of NAFLD, we differentiated four iPSC lines (Table 1) derived from donors with distinct NAFLD backgrounds into HLCs and induced fat storage by stimulation with high levels (200 µM) of oleic acid (OA).

**Table 1.**
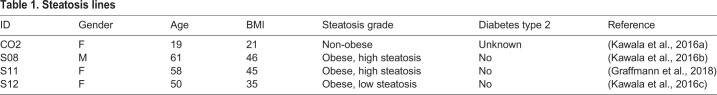
**Steatosis lines**

**Fig. 1.**
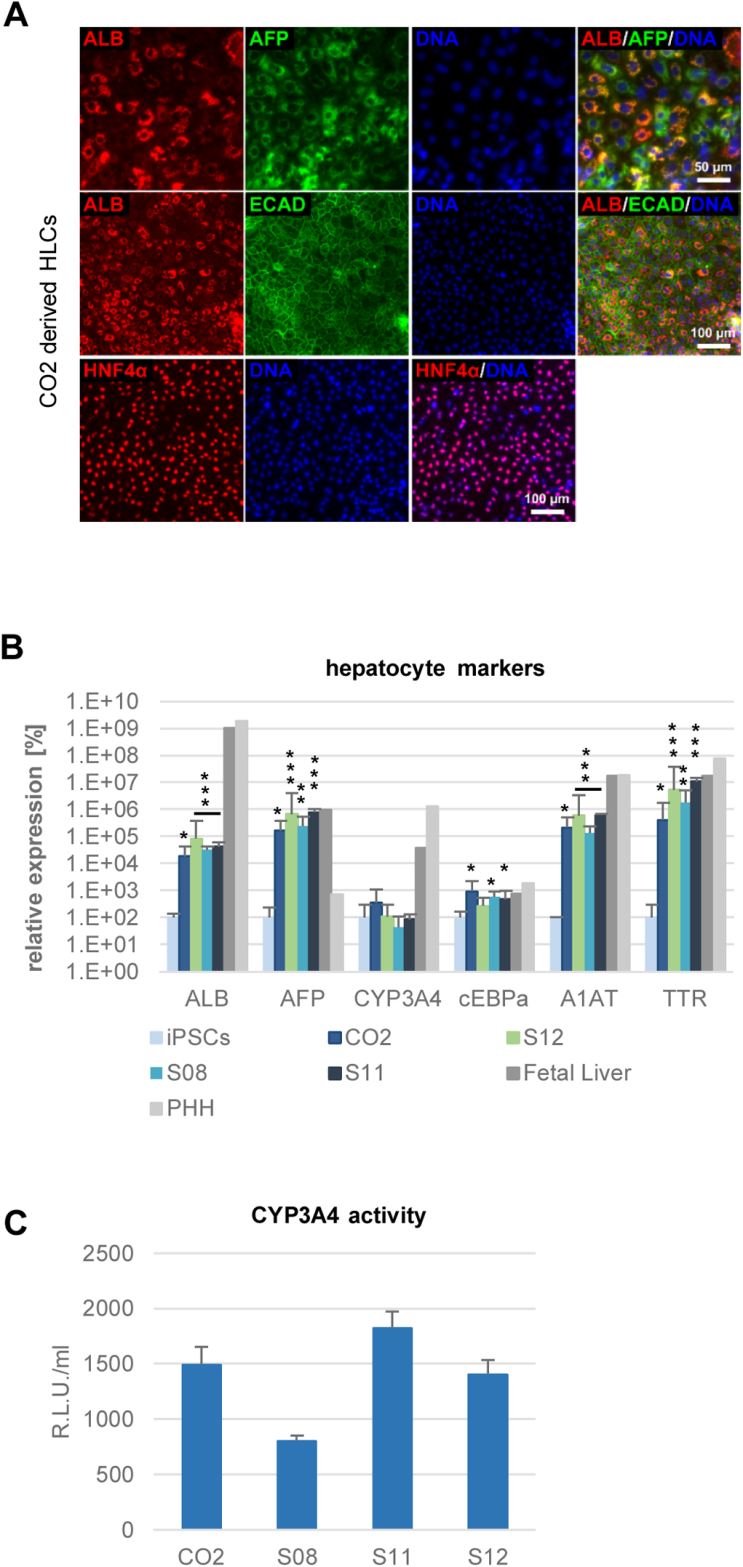
**Characterization of HLCs.** (A) Representative immunocytochemistry of hepatocyte markers at the end of HLC differentiation for the line CO2. Cells were stained for ALB (red) and AFP (green) (upper lane), ALB (red) and ECAD (green) middle lane, HNF4α (red) (lower lane). DNA was stained with Hoechst 33258. (B) Expression of hepatocyte markers *ALB*, *AFP*, *CYP3A4*, *cEBPα*, *A1AT*, and *TTR* was confirmed by qRT-PCR. Fold change towards iPSCs was calculated and converted into percentage. iPSCs: *n*=2, HLCs: *n*=3, PHH and fetal liver RNA: *n*=1. Data are means +/− 95% confidence interval. Significances in comparison to iPSCs were calculated with unpaired two-tailed Student's *t*-tests. *=*P*<0.05, **=*P*<0.01, ***=*P*<0.001. (C) CYP3A4 activity in HLCs. *n*=3, mean values +/− s.d. are shown.

The CO2 control cell line was derived from a healthy donor (Kawala et al., 2016a), while the other cell lines were generated from patients with steatosis grades between 40% and 70% (Kawala et al., 2016b,c; Graffmann et al., 2018; Wruck et al., 2015). All cell lines were efficiently differentiated into HLCs (Fig. 1; Fig. S1). Immunocytochemistry showed that the cells expressed the mature hepatocyte marker Albumin (ALB) along with the more fetal marker alpha-fetoprotein (AFP). In addition, they were positive for the epithelial marker E-cadherin (ECAD) and expressed the hepatocyte specific transcription factor hepatocyte nuclear factor 4α (HNF4α) (Fig. 1A). Comparing the expression of key hepatocyte markers in HLCs to that of iPSCs also showed significant increases (Fig. 1B). The cells expressed *AFP* in a comparable range with fetal liver cells. *ALB* expression was significantly increased in HLCs compared to iPSCs. Expression levels of two other hepatocyte specific markers, *alpha-1-antitypsin* (*A1AT*) and *Transthyretin* (*TTR*) were relatively close to that in adult liver-PHH and fetal liver and at least 1000 times higher than in iPSCs. All cell lines showed Cytochrome P450 (CYP) 3A activity, albeit on a low level (Fig. 1C), which is characteristic for *in vitro* derived HLCs.

### HLCs derived from donors with distinct grades of steatosis can store LDs after OA induction

**Fig. 2.**
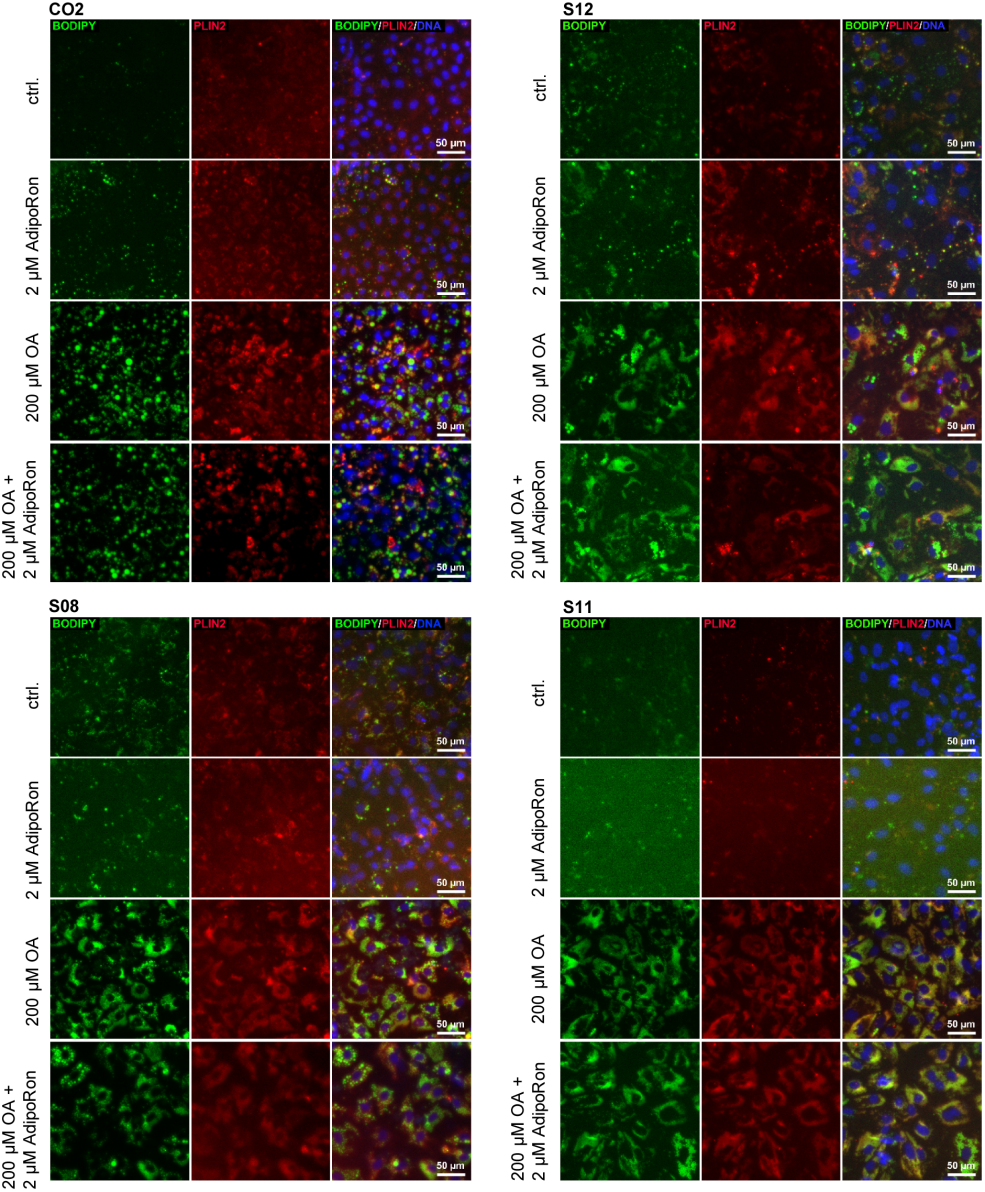
**Fat induction in HLCs.** Representative immunocytochemistry for LDs (BODIPY 493/593, green), PLIN2 (red) and DNA (Hoechst 33258, blue) in iPSC derived HLCs.

We added 200 µM OA into the medium for several days to see if all cell lines were capable of storing fat in the form of LDs. We observed a significant increase of LDs after 9 days of OA induction (Fig. 2A). All four lines had low basal levels of LDs. After induction, the amount of LDs increased in all cell lines, while the pattern was clearly different. CO2 cells formed huge and clearly separated LDs, whereas S11 cells incorporated lots of tiny LDs. Both types of LDs could be observed in S08 and S12 cells.

**Fig. 3.**
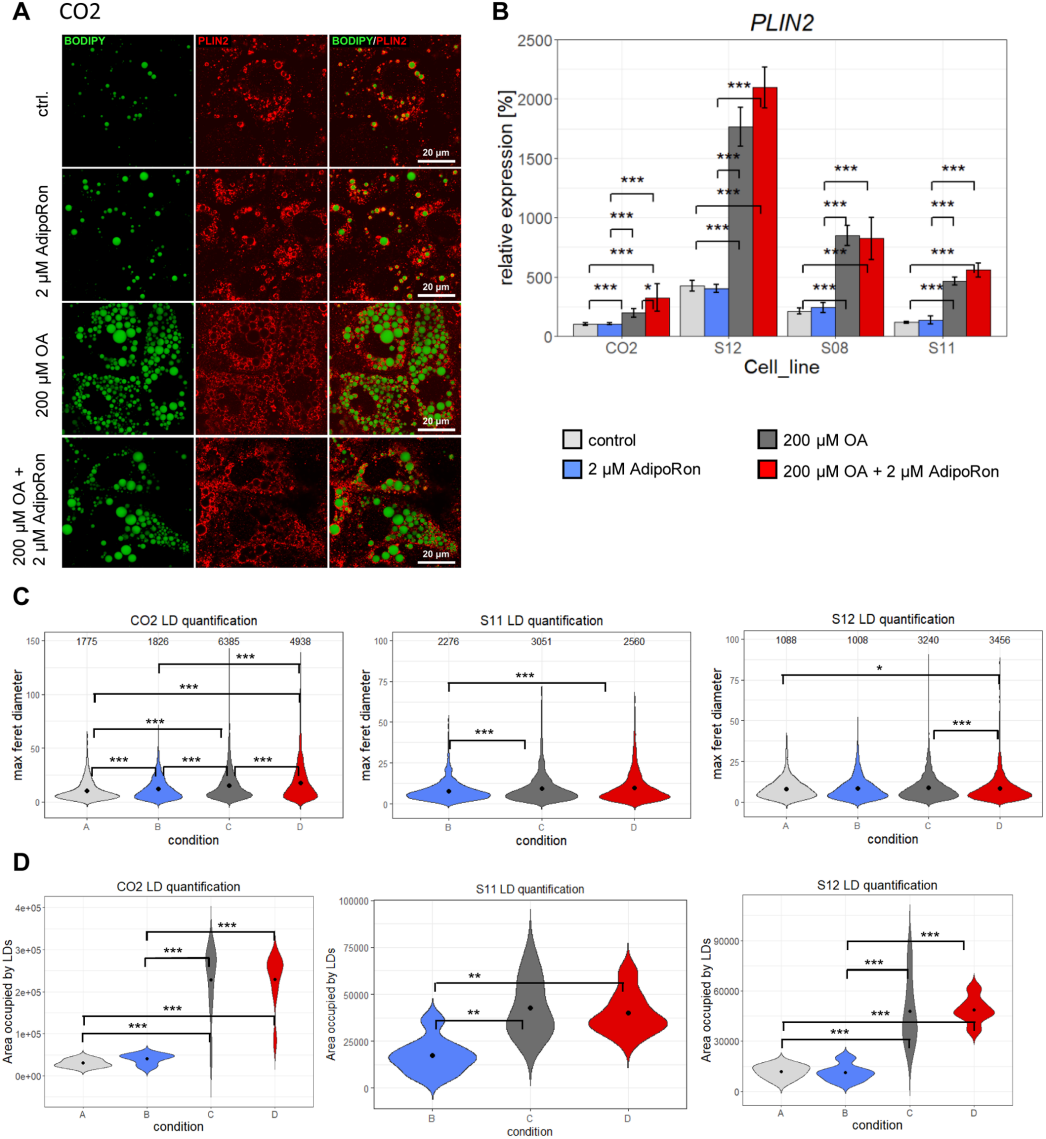
**LD quantification.** (A) Confocal microscopy of CO2 cells. LDs (BODIPY 493/593, green), PLIN2 (red). (B) *PLIN2* expression was measured by qRT-PCR. Fold change was calculated towards CO2 control cells and converted into percentage. Mean of three biological replicates +/− 95% confidence interval is shown. Significances were calculated with ANOVA, followed by Tukey’s multiple comparisons of means with 95% family wise confidence levels. Number and size of LDs as well as total area occupied by LDs were calculated via Cell Profiler 3.1.9. Due to the huge size differences of LDs, two distinct pipelines had to be used for CO2 and S11/12. Data of S08 and S11 condition A is missing due to technical issues during cell culture (C) Violin plot depicting size and number of LDs. Numbers of LDs are given within the plot. Mean values of LD size are indicated as black dots. Significances were calculated with Kruskal–Wallis test (C02: *P*<2.2e-16, S11: *P*=2.027e-05, S12: *P*=0.002377) followed by Wilcoxon rank test of means. (D) Total area occupied by lipid droplets. Mean values of LD size are indicated as black dots. Significances were calculated with ANOVA (C02: *P*=1.03e-09, S11: *P*=0.000689, S12: *P*=2.42e-06), followed by Tukey’s multiple comparisons of means with 95% family wise confidence levels. *=*P*<0.05, **=*P*<0.01, ***=*P*<0.001.

In LDs, triacylglycerides are enclosed by a monolayer of lipids which is covered with a variety of proteins. One of them is perilipin (PLIN)2, which is characteristic for growing LDs and has been associated with the development of NAFLD (Pawella et al., 2014). Initially, all cell lines expressed low levels of PLIN2, which increased after fat induction. Especially in CO2 derived cells, the immunocytochemistry confirmed that LDs are enclosed by PLIN2 (Fig. 3A, Fig. S2). qRT-PCR corroborated the significant increase of *PLIN2* expression in all cell lines after OA treatment and revealed baseline differences in *PLIN2* levels between cell lines (Fig. 3B). LD quantification via cell profiler supported the observation that number as well as size of LDs increased (Fig. 3C) after OA treatment. Importantly, the total area covered by LDs increased in all cell lines significantly after OA treatment (Fig. 3D).

### Fat storage in HLCs is not influenced by AdipoRon

The adipokine adiponectin as well as its synthetic analogue AdipoRon have many positive effects on murine metabolism, e.g. reducing gluconeogenesis, lipogenesis, and hepatic fat incorporation. We sought out to test if AdipoRon also influences LD storage and metabolism in the human iPSC-derived HLCs. To this end, we incubated HLCs for 9 days with and without 200 µM OA and added 2 µM AdipoRon to each condition. Visually, we could not observe any changes in LD number or structure in cells treated with AdipoRon compared to untreated cells (Figs 2A,3A; Fig. S2), while quantification indicated that AdipoRon induced an increase in LD size in CO2 cells independent of OA treatment and a decrease in OA treated S12 cells. Only in OA treated CO2 cells, *PLIN2* expression increased with OA treatment (Fig. 3B).

### Mediators of Adiponectin signalling are present and active in all cell lines

**Fig. 4.**
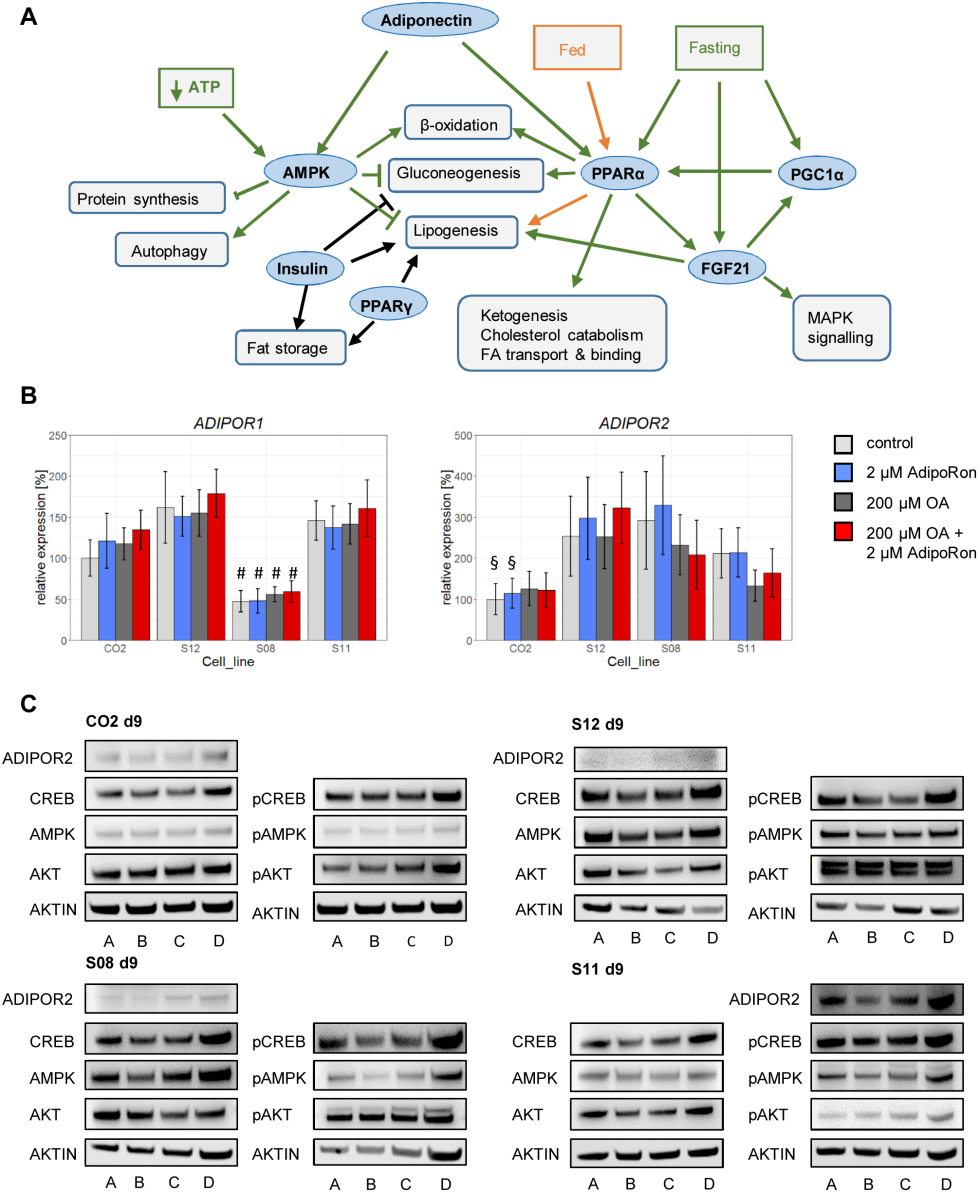
**Expression of metabolic master regulators in HLCs.** (A) Schematic overview of relevant metabolic interactions in hepatocytes. (B) qRT-PCR for *AdipoR1* and *2*. Fold change was calculated towards CO2 control cells and converted into percentage. Mean of three biological replicates +/− 95% confidence interval is shown. Significances were calculated with ANOVA, followed by Tukey’s multiple comparisons of means with 95% family wise confidence levels. #=*P*<0.001 when comparing same conditions between all lines; §=*P*<0.01 when comparing CO2 and S08 or S12. (C) Representative western blots for AdipoR2, CREB/pCREB, AMPK/pAMPK, AKT/pAKT, and β-ACTIN. A=control, B=2 µM AdipoRon, C=200 µM OA, D=200 µM OA+2 µM AdipoRon.

Since AdipoRon treatment apparently had no effect on fat storage in HLCs, we tested if the relevant pathways, which are supposed to be influenced by AdipoRon (Fig. 4A), are actually active in HLCs.

Therefore, we first analysed the expression of the adiponectin receptors AdipoR1 and 2 in all cell lines. On the mRNA level, both receptors were present in all lines and their expression was neither influenced by OA nor by AdipoRon treatment (Fig. 4B). Interestingly, *AdipoR1* expression was significantly lower in S08 HLCs than in all other lines, independent of treatment. *AdipoR2* expression tended to be lower in CO2 cells. While both receptors were expressed in all of our cells on the mRNA level, only AdipoR2, which has been described to be the major adiponectin receptor on hepatocytes (Yamauchi et al., 2003), could be detected by western blotting (Fig. 4C).

We next wanted to know if the enzymes involved in the major signalling pathways that are influenced by AdipoRon are present in the cells. Therefore, we performed western blotting for cAMP response element-binding protein (CREB), the enzyme 5′ adenosine monophosphate-activated protein (AMPK), and protein kinase beta (AKT), probing for the total protein as well as for the respective phosphorylated active forms.

In all lines, these proteins as well as their active phosphorylated counterparts were present, although with major variations between lines (Fig. 4C; Fig. S3).

### Key metabolic master regulators are expressed in HLCs

We next performed qRT-PCR to see whether key metabolic regulators are expressed in our cells and how they react to the OA challenge and the AdipoRon treatment. Of special interest were the peroxisome proliferator-activated receptor (PPAR) family members *PPARα* and *y*, as well as Protein Kinase AMP-Activated Catalytic Subunit Alpha (PRKAA)2, the catalytic subunit of AMPK.

**Fig. 5.**
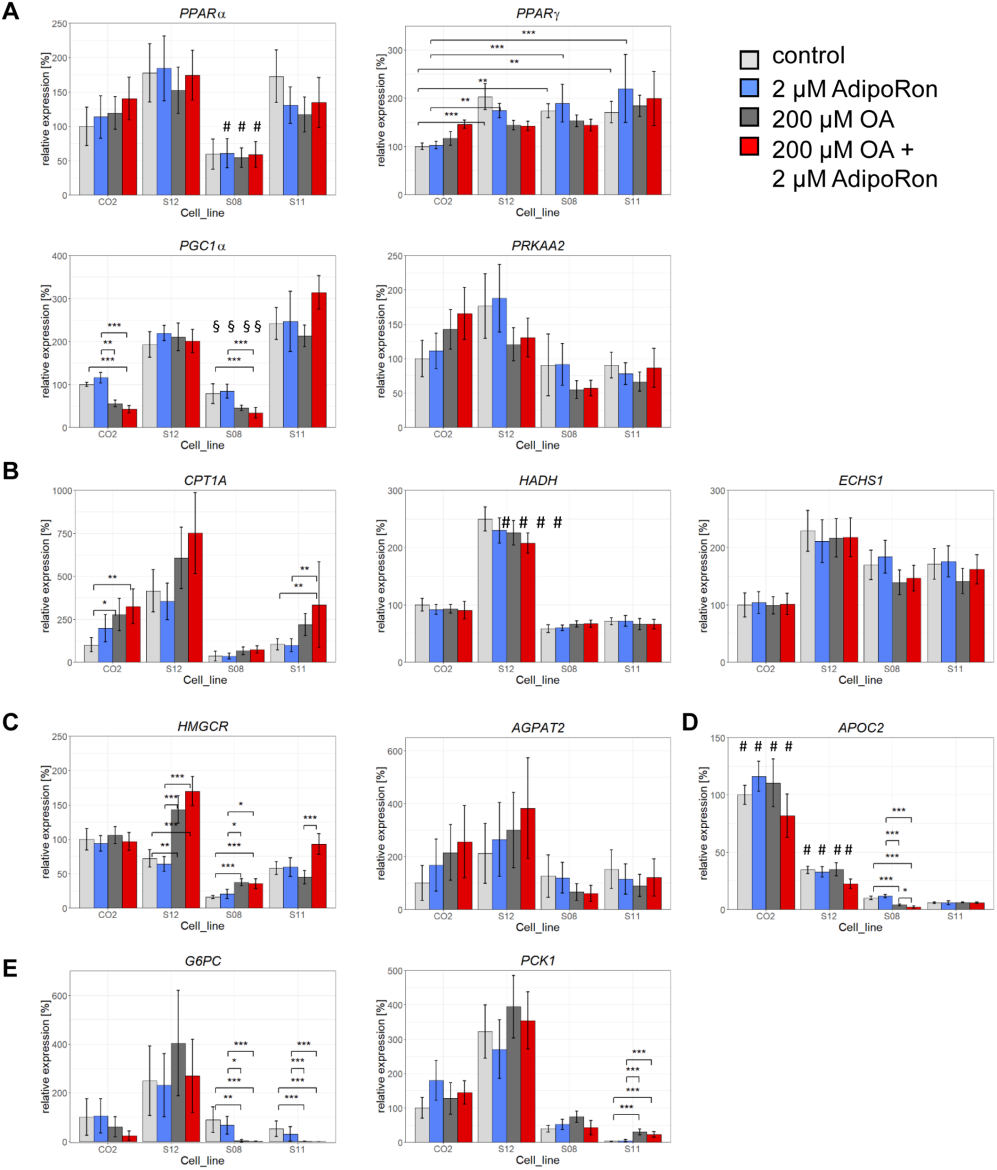
**Differential expression of metabolic enzymes.** qRT-PCR for enzymes involved in metabolic regulation (A): *PPARα* (#=*P*<0.05 when comparing same conditions between all lines), *PPARγ*, *PGC1α* (§=*P*<0.001 when comparing same conditions between S08 and S11 or S12; $=*P*<0.001 when comparing same conditions between S11 and S08 or CO2), *PRKAA2*, in β-oxidation (B): *CPT1A*, *HADH* (#=*P*<0.001 when comparing same conditions between all lines), *ECHS1*, in lipid and cholesterol metabolism (C): *HMGCR*, *AGPAT2*, in lipid export (D): *APOC2* (#=*P*<0.001 when comparing same conditions between all lines), in gluconeogenesis (E): *G6PC*, *PCK1*. Fold change was calculated towards CO2 control cells and converted into percentage. Mean of three biological replicates +/− 95% confidence interval is shown. Significances were calculated with ANOVA, followed by Tukey’s multiple comparisons of means with 95% family wise confidence levels. *=*P*<0.05, **=*P*<0.01, ***=*P*<0.001.

Besides being involved in Adiponectin signalling, it is known that hepatic PPARα gets activated by fatty acids that are released from adipocytes. It stimulates energy generating metabolic pathways, in particular β-oxidation (Pawlak et al., 2015). Here, we did not observe any substantial changes in *PPARα* expression related to OA or AdipoRon treatment. Interestingly, S08 cells had a significantly lower expression of *PPARα* with and without challenge than all other lines (Fig. 5A).

PPARy is known to increase fat storage (Medina-Gomez et al., 2007). At baseline as well as with 2 µM AdipoRon treatment alone, its expression was significantly lower in CO2 derived HLCs than in all other lines. Overall, we did not observe expression changes related to OA or AdipoRon treatment (Fig. 5A).

PPARy Coactivator-1α (PGC1α) is a transcriptional coactivator that interacts, amongst others, with PPARα and γ. It is involved in the upregulation of gluconeogenesis genes during fasting as well as in the induction of β-oxidation. It is known that, in the fed state, PGC1α is expressed at low levels in the liver and that expression increases during fasting (Yoon et al., 2001). In our setting, *PGC1α* was generally expressed at lower levels in CO2 and S08 cells than in S11 and S12. In the lines that expressed *PGC1α* at low levels, the expression was even further reduced after OA treatment independent of AdipoRon (Fig. 5A).

Finally, to assess AMPK levels, we measured *AMPK Subunit Alpha-2* (*PRKAA2*) expression. Apart from its role in Adiponectin signalling, AMPK acts as a sensor of nutritional levels and reduces gluconeogenesis while it increases β-oxidation. After OA induction, *PRKAA2* expression was reduced in all cell lines except CO2, although the effect was not significant (Fig. 5A).

### Enzymes involved in fatty acid and cholesterol metabolism are differentially expressed

To see if OA induction or AdipoRon treatment have any effects on downstream metabolic enzymes, we assessed the expression of lipid metabolism associated genes, which was strikingly different between cell lines. First we looked at genes involved in mitochondrial β-oxidation. Carnitine Palmitoyltransferase 1A (CPT1A) is the rate limiting enzyme responsible for the transport of fatty acid derived acyl-CoA across the mitochondrial membrane. In general, its expression was lower in the high steatosis lines S08 and S11 than in the low steatosis line and the control line. Interestingly, we observed a significant increase of *CPT1A* expression in CO2 and S11 cells after induction with OA alone as well as in combination with AdipoRon (Fig. 5B).

In case of Hydroxyacyl-CoA Dehydrogenase (*HADH*), which is involved in mitochondrial β-oxidation, we observed strikingly high expression levels in S12 cells in all conditions, while for Enoyl-CoA Hydratase Short Chain 1 (*ECHS1*), which also is important for this process, CO2 cells expressed remarkably low levels. For both factors, we could not observe expression changes related to OA or AdipoRon (Fig. 5B).

We also analysed the expression of genes important for cholesterol and lipid synthesis. 3-Hydroxy-3-Methylglutaryl-CoA Reductase (HMGCR) is involved in cholesterol synthesis. Its expression levels varied markedly between cell lines, with the lowest levels in S08 and S11 cells. Its expression was significantly upregulated in the high steatosis line S08 and the low steatosis line S12 after OA treatment independent of AdipoRon. Only in S11 cells, treatment with 2 µM AdipoRon significantly increased *HMGCR* expression in the OA condition (Fig. 5C).

Similar to *HMGCR*, the expression of 1-Acylglycerol-3-Phosphate O-Acyltransferase 2 (*AGPAT2*), which plays a role in phospholipid biosynthesis, was highly variable in all cell lines, with S08 and S11 expressing the lowest levels of *AGPAT2* (Fig. 5C).

Finally, we analysed the expression of Apolipoprotein C2 (*APOC2*), which is involved in coating of very low-density lipoproteins (VLDL) that are secreted into the blood. Here, we observed in all conditions three to ten times higher expression levels in CO2 cells than in all other lines. We observed a significant reduction of APOC2 expression only in S08 cells, after OA treatment, this was even further reduced upon AdipoRon stimulation (Fig. 5D).

### OA treatment influences gluconeogenesis

We also wanted to know, whether there are differences in our lines with regards to the regulation of gluconeogenesis. In this regard, we analysed the expression of key genes involved in this process. Glucose-6- phosphatase (G6PC) is part of the catalytical complex that hydrolyses glucose 6-phosphate to glucose, the last step during gluconeogenesis. Its expression levels were generally low in all cell lines except S12. *G6PC* expression was significantly reduced in all lines except S12 after OA induction (Fig. 5E). Phosphoenolpyruvate Carboxykinase 1 (PCK1) catalyses the rate limiting step of gluconeogenesis, the transformation of oxaloacetate to phosphoenolpyruvate. Its expression was for all conditions highest in the low steatosis lines CO2 and S12, while it was almost undetectable in untreated S11 cells (Fig. 5E).

Taken together, the variations in the PCR data suggest the existence of cell type associated gene expression patterns that obscure the effects of OA and AdipoRon treatments at the given concentrations. Probably, a more stringent experimental approach, including age, gender and disease stage matched cells as well as a higher AdipoRon concentration will be necessary to unambiguously reveal metabolic patterns.

Nonetheless, we could identify a steatosis related phenotype (Table 2) with the high steatosis lines S11 and S08 tending to have low expression of genes involved in lipid export, fat and cholesterol synthesis as well as in gluconeogenesis, β-oxidation and FGF21 signalling.

**Table 2.**
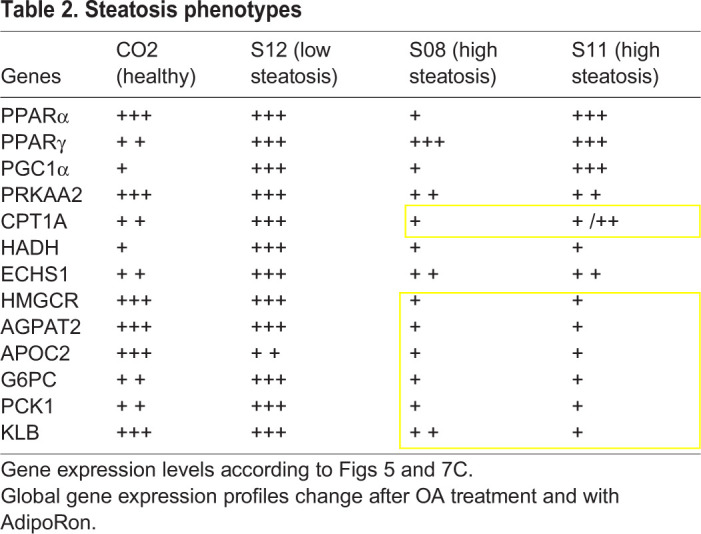
**Steatosis phenotypes**

All analyses indicated a more prominent role for OA regarding gene expression changes than for AdipoRon, at least in the selected pathways. To reveal any AdipoRon associated gene expression patterns, we performed Affymetrix Clariom S Microarray analyses for CO2 samples with and without treatment. As we saw a lot of variability in the PCR data, we restricted the microarray analysis to the cell line which has been generated from a healthy control donor in order to minimalize cell line dependent or culture induce effects in the results.

**Fig. 6.**
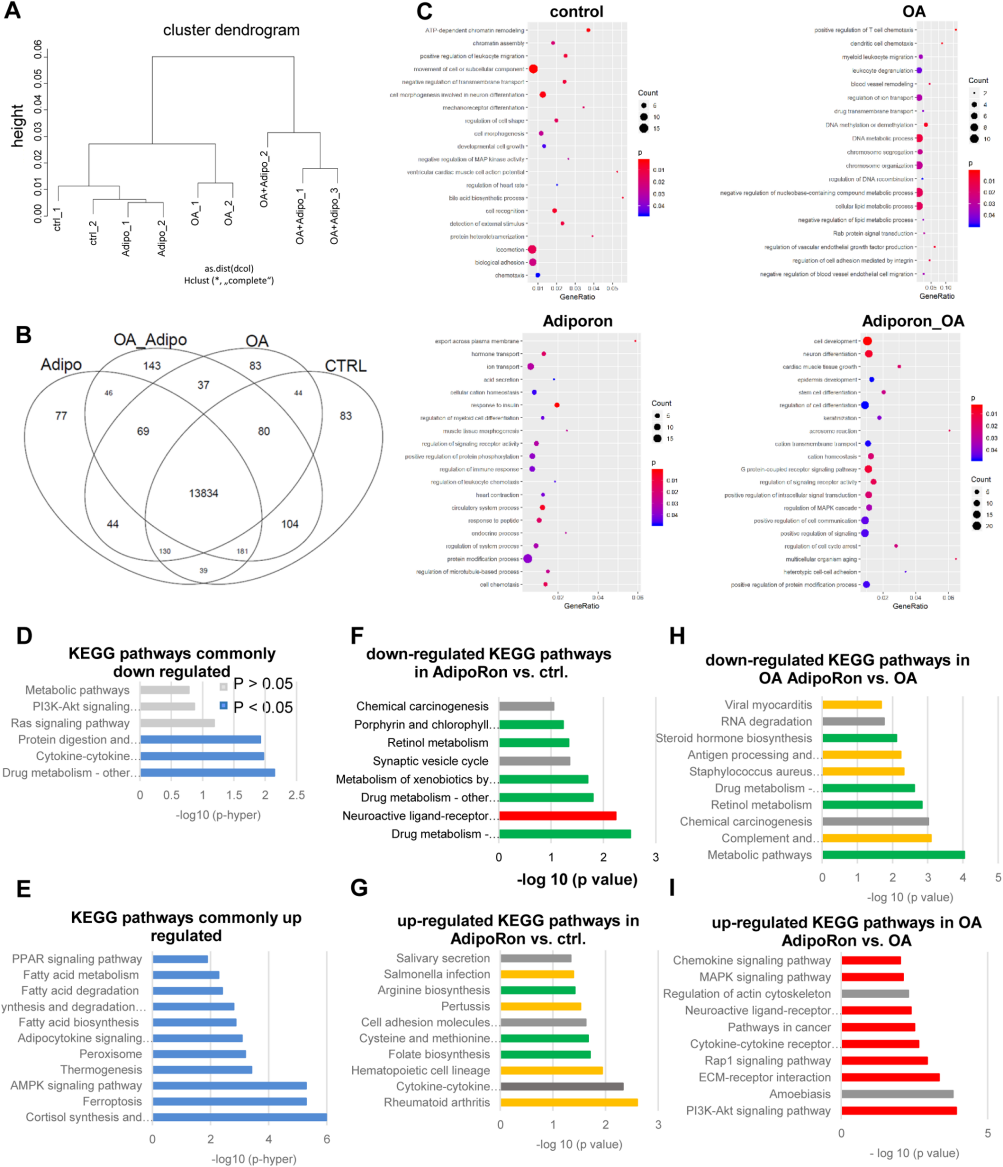
**Changes of global gene expression profiles after OA treatment and with AdipoRon.** Transcriptome analysis was performed for all four conditions of CO2 HLCs. (A) Cells cluster according to treatment. (B) Venn diagram depicting the exclusively expressed genes for all four conditions. (C) Selected significantly enriched GOs of the exclusively expressed genes in the indicated conditions. (D,E) Comparison with published data of differentially expressed genes in iPSCs derived HLCs after OA treatment reveals common downregulated (D) and selected common upregulated (E) KEGG-pathways. (F–I) Top 10 significantly down- or upregulated KEGG pathways after AdipoRon treatment of control and OA cells. For full lists of GOs and KEGG pathways please refer to Table S1.

Global analysis of gene expression revealed four distinct clusters, according to the four treatments (Fig. 6A). Overall, 13,834 genes were expressed in common in CO2 HLCs, independent of treatment (Fig. 6B). For every condition, we identified the exclusively expressed genes by Venn diagram analysis (Fig. 6B). 77 were only expressed in AdipoRon treated cells, 143 in cells treated with AdipoRon and OA, 83 in the OA only cells as well as in the untreated control cells. These exclusively expressed genes were related to distinct gene ontologies (GOs), indicating specific profiles of the 4 treatments (Fig. 6C). No characteristic GOs were associated with control cells. OA treated cells, on the other hand, exclusively expressed numerous genes associated with DNA replication/repair, immune reactions and metabolism. AdipoRon treatment of OA cells induced genes involved in signalling, while in the control condition, AdipoRon predominantly influenced metabolism-associated genes. For the full lists of GOs, please refer to Table S1.

In order to check the robustness of our model, we compared the differentially expressed genes between OA treated and control cells with those identified in a previous study also using iPSCs as a model for NAFLD (Graffmann et al., 2016) (Fig. 6D,E). There was an overlap of 24 genes upregulated and 32 genes downregulated after OA treatment. KEGG pathway analysis revealed that the common downregulated genes were significantly associated with drug metabolism, cytokine–cytokine receptor interaction and protein digestions, while signalling and metabolic pathways were also detected although this was not significant (Fig. 6D). Importantly, the common significantly upregulated pathways were predominantly associated with metabolism as well as with adipocytokine and AMPK signalling (Fig. 6E).

Next, we checked which KEGG pathways were affected by AdipoRon treatment in the control and OA setting. In the control treatment, AdipoRon mostly affected metabolism and immune system related pathways. Interestingly, drug metabolism tended to be downregulated while metabolic pathways related to amino acid synthesis as well as pathways related to the immune system, were upregulated (Fig. 6F,G).

On the OA background, pathways related to metabolism and immune system were downregulated (Fig. 6H). The upregulated pathways in OA AdipoRon-treated cells were predominantly related to various signalling pathways (Fig. 6I).

In order to identify an AdipoRon-associated signature, we compared the common up- and downregulated genes in AdipoRon-treated control and OA cells (Fig. S4). Among the significantly upregulated pathways, we identified transmembrane transporters, drug metabolism, and glycoprotein/thyroid hormones. The significantly commonly downregulated pathways were connected to homeostasis, indicating a broad role for AdipoRon on metabolism and cell function in general.

### FGF21 expression is reduced after OA treatment

**Fig. 7.**
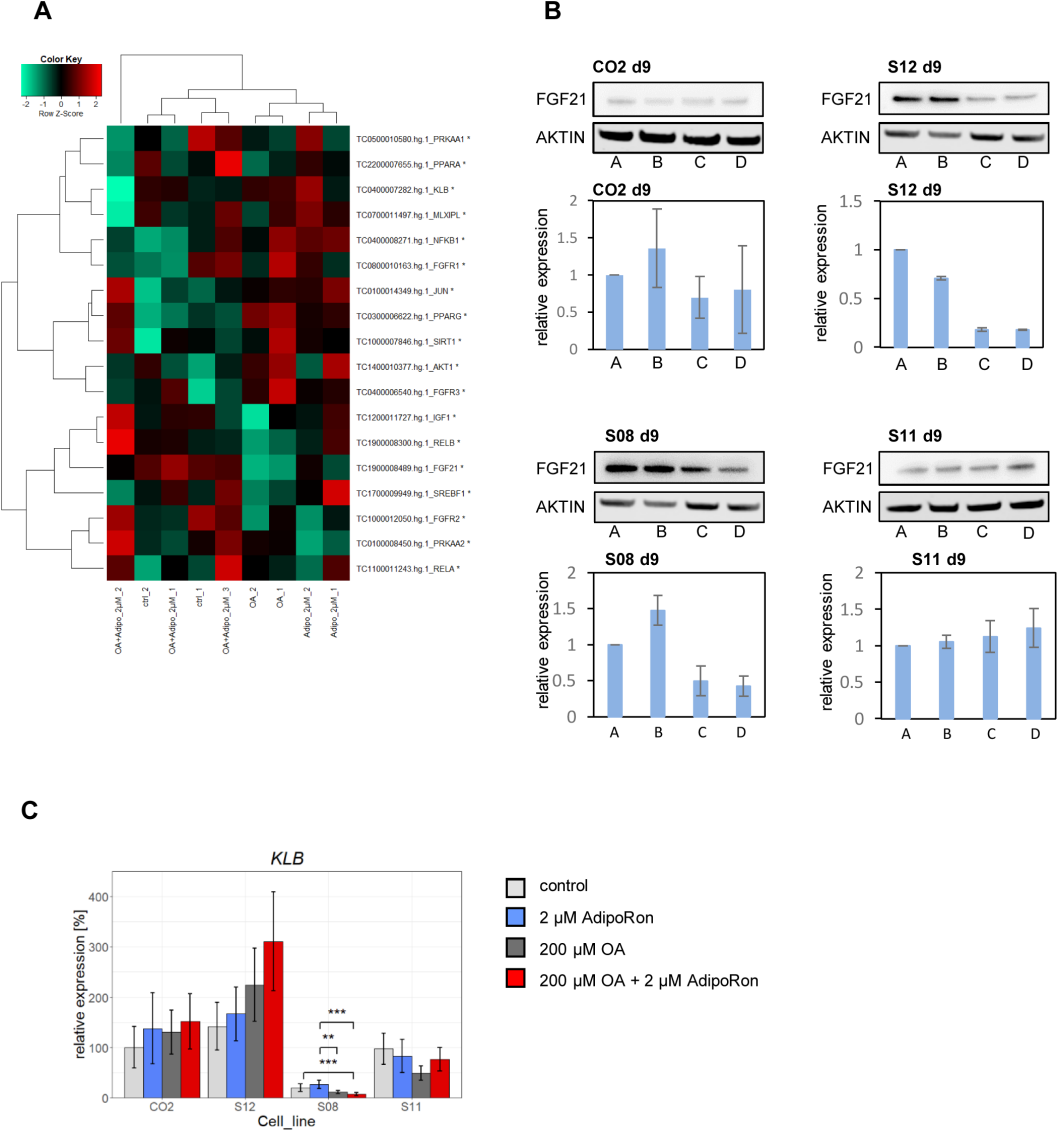
**FGF21 expression changes by OA treatment.** (A) Heatmap analysis of genes within the Adiponectin-PPARα metabolic network. (B) Representative western blots of three independent blots for FGF21 and β-ACTIN and quantification of FGF21 expression, normalized to control conditions. *n*=3, mean±s.d. is shown. A=control, B=2 µM AdipoRon, C=200 µM OA, D=200 µM OA + 2 µM AdipoRon. (C) Expression of KLB was measured by qRT-PCR. Fold change was calculated towards CO2 control cells and converted into percentage. Mean of three biological replicates +/− 95% confidence interval is shown. Significances were calculated with ANOVA, followed by Tukey's multiple comparisons of means with 95% family wise confidence levels. **=*P*<0.01, ***=*P*<0.001.

Finally, we selected genes of the metabolic network involved in PPARα and Adiponectin signalling (Fig. 4A) for heatmap analysis. Interestingly, *FGF21* expression was downregulated in OA-treated cells compared to control cells (Fig. 7A,B). FGF21 acts as a hormone in an endocrine, autocrine and paracrine manner and is tightly associated with Adiponectin and PPARα/γ signalling (Lin et al., 2013; Goto et al., 2017; Gälman et al., 2008). FGF21 is predominantly synthesized in the liver. Its expression is regulated by PPARα and γ. In turn, FGF21 can regulate Adiponectin as well as PPARγ expression in feed-forward-loops (Goetz, 2013). For all cell lines except of S11 we could confirm the OA-associated reduction of FGF21 expression in western blots.

FGF21 signals via receptor dimers consisting of various FGF receptors in combination with β-KLOTHO (KLB). The common factor for signalling, *KLB*, was expressed in all cell lines independent of treatment. Similar to *AdipoR1*, its expression was significantly reduced in S08 cells (Fig. 7C).

In summary, we have shown that *in vitro* derived HLCs from various donors with distinct genetic backgrounds react similarly to OA overdose with incorporating fat and increasing *PLIN2* expression. Apart from that, there are marked differences in the gene expression profiles of the different cell lines reflecting the complex metabolic pathways that seem to play varying roles in the individual lines and could explain the differences seen in disease progression within individuals. While we could not identify a robust AdipoRon effect on an isolated factor, we saw general metabolic alterations affecting metabolism, transport, and signalling pathways.

## DISCUSSION

NAFLD is a multifactorial disease that is regulated by complex interactions between genome, epigenome, and microbiome in response to certain nutritional cues. Here, we employed an iPSC based *in vitro* model for NAFLD to assess a variety of phenotypes associated with the disease.

All our iPSC-derived HLCs from different donors accumulated LDs in response to a high fat diet. We saw substantial differences in the quantity, size, and distribution of LDs in all four cell lines, while all of them significantly upregulated *PLIN2*, a crucial LD-coating protein, in response to OA treatment. Interestingly, the cells that were derived from the healthy control donor produced the biggest LDs which even increased after AdipoRon treatment. In parallel, *PLIN2* expression levels after OA induction were lower than in all other cell lines. S11 cells, which were derived from a high steatosis patient, accumulated an uncountable amount of very tiny LDs. Also here, *PLIN2* expression was relatively low. Strikingly, S12 cells, which were derived from a low steatosis donor and showed an intermediate phenotype regarding LD size and quantity, had the highest induction of *PLIN2*. While the specific morphologies and distribution of LDs might be associated with disease severity, further investigations comparing several high-steatosis patient and healthy donor derived samples are necessary to exclude influences of age, gender, and cell culture effects.

In humans, macrovesicular steatosis, where few big LDs are formed, has a less negative impact on liver function and whole body health than microvesicular steatosis, which often is accompanied by encephalopathy and liver failure (Tandra et al., 2011). The phenotype of OA-fed CO2 cells mimics that of macrosteatosis. Low levels of PLIN2 are associated with a lean phenotype and a reduced risk for steatosis in mice (McManaman et al., 2013). The combination of large LDs with relatively low levels of *PLIN2* expression in CO2-derived HLCs could point towards a yet unknown mechanism that protects the cells from lipid induced damage, which might be enhanced by AdipoRon treatment.

Additional indications of a healthier phenotype in CO2 cells are given by its relatively high expression of *CPT1A* and *APOC2*, possibly related to efficient burning and export of fatty acids. In contrast, gene expression patterns in the high steatosis lines indicate impaired fasting responses with low levels of *PPARα* in S08 cells and no changes in *PGC1α* after OA induction in S12, S11 and S08 cells. In addition, these cells seem to have an impaired capability of exporting FAs as suggested by the low levels of *APOC2* expression.

By integrating these data, we were able to identify critical metabolic constellations that suggested a more severe steatosis phenotype. High steatosis lines had a rather low expression of genes associated with gluconeogenesis, phospholipid-, and cholesterol biosynthesis with concomitant low expression of *CPT1A* indicating an additional lower capacity of β-oxidation and thus energy generation (Table 2).

Interestingly, all cells except S11 had reduced FGF21 levels after OA treatment. Normally, hepatic FGF21 expression is related to the fasting response (Gälman et al., 2008; Inagaki et al., 2007), thus low levels of FGF21 after OA overfeeding could be expected. Thus, the failure to reduce FGF21 levels in response to OA could be an additional sign of inefficient metabolic regulation in S11 cells. Interestingly, levels of PPARα, which enhance FGF21 expression, and levels of KLB, which transfer FGF21 signalling into the cell, are within the range of the other cell lines and thus do not seem to be responsible for the failure to regulate FGF21 levels.

Taken together, our data point to an impaired reaction to nutritional cues in HLCs derived from high steatosis patients. Further comparative analysis will show if these cells really produce less glucose while also generating less energy which overall could be related to a limited capability to match the bodies energy needs which could trigger a compensatory storage of fat.

Overall, many aspects of NAFLD can be recapitulated *in vitro*, independent of the donor's genotype. However, the distinct origin of the cells and their metabolic capacities, as well as distinct reprogramming and differentiation efficiency, have a key impact on the analyses and impede unambiguous conclusions at this stage.

In general, OA treatment had major effects on the cells, while AdipoRon effects only became visible when analysing whole transcriptome data from one single donor. Possibly, its influence might become more obvious by increasing the concentration or duration of AdipoRon treatment and including more replicates in every analysis.

The transcriptional network that regulates key metabolic processes and is supposed to be susceptible to Adiponectin signalling was active in all cells. They all expressed AdipoR2 as well as AMPK, CREB, and AKT, which were all also detectable in the phosphorylated, active form. However, we did not see reproducible disease-associated phenotypes and we were also not able to induce consistent changes in the activity levels of the analysed regulators by OA or AdipoRon treatment. This might be due to the complex interaction of several pathways and the simultaneous presence of conflicting signals that are present in the cell culture. The HLC medium contains for example insulin as well as the glucocorticoid dexamethasone which both are strong inductors of fat storage (Brown and Goldstein, 2008; Marino et al., 2016). We do not know if cells from all donors react in the same way to these molecules. Maybe higher AdipoRon concentrations are necessary to induce beneficial metabolic effects in all cell lines. In addition, it is possible that some AdipoRon related effects become only obvious in the systemic setting and cannot be reproduced in an *in vitro* model.

When analysing only the CO2 cell line, we observed influences of OA and AdipoRon on the transcriptome. The cells clustered according to the treatment. Comparison of the up- and downregulated genes after OA treatment with previously generated and published data from our system (Graffmann et al., 2016) revealed 56 overlapping genes. This number is somewhat limited due to different cell lines that were used and differences in the OA induction protocol. Nonetheless, there are commonly regulated genes. These are probably reliable as indicators for a steatotic phenotype because they were regulated in a robust way across the experiments. Interestingly, in both studies PPAR- and AMPK signalling as well as fat metabolism were upregulated, suggesting a common reproducible pattern. Especially PPAR-signalling pathways are already clinical targets for treating hyperlipidemia. So far, these medications are not approved for the treatment of NAFLD but our data support studies that claim efficiency of PPAR, agonists in this condition (Boeckmans et al., 2019; Fernandez-Miranda et al., 2008).

Analysis of the genes exclusively expressed in the four conditions revealed distinct patterns of overrepresented GOs. Most importantly, AdipoRon influenced metabolism-associated GOs in the control setting while it had an impact on signalling in the OA background. OA treatment alone induced stress in the cells, which becomes evident by many of the upregulated GOs associated with DNA repair and structure as well as to the immune system. Increased cellular stress levels are tightly connected to the progression of NAFLD to NASH and HCC (Buzzetti et al., 2016).

AdipoRon seems to have distinct functions depending on the nutritional background. As expected, it is involved in the regulation of metabolism in the control as well as in the OA setting. Interestingly, in the control AdipoRon condition, several pathways related to cysteine, methionine and folate metabolism were upregulated. Indeed, deprivation of cysteine and methionine fosters the development of NASH in mice (Rinella et al., 2008), which might be counteracted by AdipoRon. AdipoRon also influenced several pathways that are connected to the immune system, which agrees with recent publications that have described an anti-inflammatory role of Adiponectin in cardiac and adipose tissue, which also was connected to milder inflammation levels in the context of the metabolic syndrome (Jenke et al., 2013; Tsuchida et al., 2005; Frühbeck et al., 2017). Also this might help to improve health conditions of steatotic patients, as latent inflammation is a risk factor for disease progression (Tilg and Moschen, 2010). Finally, AdipoRon increased signalling pathways, many of which are involved in regulating metabolism, in OA treated cells. Although we could not confirm the AdipoRon action in the selected pathways in our analysis, these data point to a global role of AdipoRon affecting metabolism. It is possible that higher concentrations of AdipoRon might give a clearer picture of its action. In addition, certain limitations of the cell culture setting probably also obscure AdipoRon effects. In 2D cultures, HLCs only reach limited grades of maturation, resembling fetal rather than adult cells which certainly has an impact on their metabolism. Also, differentiation efficiency varies between cell lines, introducing additional variability when comparing cells from distinct donors (Hannan et al., 2013). Recently, 3D culture models have been published, which increase maturity and might be suitable to overcome the problem of varying differentiation efficiencies (Rashidi et al., 2018; Sgodda et al., 2017). Although in this setting we face the question whether or not externally applied substances reach all cells, especially those inside the organoid, a more homogenous culture might nonetheless improve our insights into NAFLD development and metabolic regulation by AdipoRon.

Despite its limitations, the heterogeneity which we find in our cell culture samples should be taken into account when developing treatments for NAFLD patients. Although there probably exist common pathways that can be modified, every patient might react differently and personalized medicine is necessary to effectively treat this widespread disease.

## MATERIALS AND METHODS

### Differentiation of iPSCs into HLCs

The use of iPSC lines for this study was approved by the ethics committee of the medical faculty of Heinrich-Heine University under the number 5013. iPSCs were cultured on laminin (LN) 521 (Biolamina) coated plates in StemMACS iPSC brew medium (Miltenyi). Differentiation into HLCs was performed as described previously (Graffmann et al., 2016) with minor changes. To start the differentiation, iPSCs were passaged as single cells onto plates coated with a 3:1 mixture of LN111 and LN521. The next day, the medium was changed to definitive endoderm (DE) medium: 96% RPMI 1640, 2% B27 (without retinoic acid), 1% Glutamax (Glx), 1% Penicillin/Streptomycin (P/S) (all Gibco), 100 ng/ml Activin A (Peprotech), which was replaced daily. On the first day an additional 2.5 µM Chir 99021 (Stemgent) was included. Afterwards the cells were cultivated for 4 days in hepatic endoderm (HE) medium with daily medium changes: 78% Knockout DMEM, 20% Knockout serum replacement, 0.5% Glx, 1% P/S, 0.01% 2-Mercaptoethanol (all Gibco) and 1% DMSO (Sigma-Aldrich). In the last step, hepatocyte-like medium was used for up to 10 days with medium change every other day: 82% Leibovitz 15 medium, 8% fetal calf serum, 8% Tryptose Phosphate Broth, 1% Glx, 1% P/S (all Gibco) with 1 µM Insulin (Sigma-Aldrich), 10 ng/ml hepatocyte growth factor (HGF) (Peprotech), 25 ng/ml Dexamethasone (DEX) (Sigma-Aldrich).

### Synthesis of AdipoRon

AdipoRon was synthesized from 4-hydroxy-benzophenone, chloroacetic acid methyl ester, and 4-amino-1-benzylpiperidine following the procedure reported by Okada-Iwabu, Yamauchi, and Iwabu (Okada-Iwabu et al., 2013; Kadowaki et al., 2015). The identity and purity of the product was double-checked by spectroscopic analysis (^1^H NMR and ^13^C NMR).

### Fat induction and small molecule treatment

Oleic acid (Calbiochem) was bound to fatty acid free BSA (Sigma-Aldrich) and added to the cells in a final concentration of 200 µM. AdipoRon was dissolved in DMSO and the cells were treated with a final concentration of 2 µM. Control treatment for OA consisted in BSA and for AdipoRon in DMSO. The treatment started on day 10 of the differentiation and was continued for 5 and 9 days.

### Immunocytochemistry

Cells were fixed with paraformaldehyde for 15 min at room temperature (RT). For permeabilization and blocking they were incubated for 2 h at RT with blocking buffer (1× PBS with 10% normal goat or donkey serum, 1% BSA, 0.5% Triton and 0.05% Tween). Blocking buffer was diluted 1:2 with 1× PBS and cells were incubated with the primary antibody overnight at 4°C. Cells were washed three times with 1x PBS/ 0.05% Tween and incubated with the secondary antibody for 2 h at RT. To stain lipid droplets, cells were incubated with BODIPY 493/503 (1 µg/ml, Life Technologies) in PBS/0.05% Tween for 20 min and washed afterwards. DNA was stained with Hoechst 33258. Images were captured using a fluorescence microscope (LSM700, Zeiss). The following primary antibodies were used: Alpha Fetoprotein, Albumin (Sigma-Aldrich), E-cadherin (CST), HNF4α (Abcam), SOX17 (R&D), PLIN2 (Proteintech). For details on antibodies see Table S2. Individual channel images were processed and merged with Fiji.

### LD quantification

For confocal images, cells were differentiated on matrigel coated x-well tissue culture chambers (Sarstedt), except for S08, where iPSCs did not attach to the glass bottom. Similarly, one condition of S11 was lost due to attachment issues. Confocal images were analysed with Cell Profiler version 3.1.9. Due to the huge differences in LD size, separate pipelines had to be used for CO2 and S11/12 analysis. Pipelines are available upon request. Significances for LD size and numbers were calculated via Kruskal–Wallis test followed by Wilcoxon rank test and for total area occupied by ANOVA followed by Tukey’s multiple comparisons of means with 95% family wise confidence levels.

### Measurement of cytochrome P450 activity

The P450-Glo^TM^ CYP3A4 Assay Luciferin-PFBE (Promega) kit was used to measure Cytochrome P450 3A4 activity employing a luminometer (Lumat LB 9507, Berthold Technologies).

### Western blot

Frozen cell pellets were lysed in 1x RIPA buffer (Sigma-Aldrich) with protease and phosphatase inhibitors (Roche, Sigma-Aldrich). 20 µg of protein were loaded into nupage 4–12% bis-tris precast gels (Thermo Fisher Scientific) and run with MES buffer. Proteins were transferred to a 0.45 µm nitrocellulose membrane (GE healthcare). Membranes were blocked with 5% milk in TBS/0.1% Tween (TBST) for 1 h at RT. Antibodies were diluted as described in Table S2. Incubation with primary antibodies was performed overnight at 4°C. Membranes were washed three times with TBST and secondary antibody incubation was performed for 1–2 h at RT followed by washing as above. In case of HRP coupled secondary antibodies, chemiluminescence was detected on a Fusion FX instrument (PeqLab). For detection of β-actin an IR dye 680 coupled secondary antibody (LICOR) was used and detection was performed on an Odyssey CLx instrument (LI-COR). Analysis was performed with Fusion Capt Advance software (PeqLab) using rolling ball background correction or with Image Studio light 5.2 software (LI-COR).

### RNA isolation and quantitative reverse transcription PCR (qRT-PCR)

Cells were lysed in Trizol. RNA was isolated with the Direct-zol™ RNA Isolation Kit (Zymo Research) according to the user's manual including a 30 min DNase digestion step. 500 ng of RNA were reverse transcribed using the TaqMan Reverse Transcription Kit (Applied Biosystems). Primer sequences are provided in Table S3. All primers were ordered from MWG.

Real time PCR was performed in technical triplicates with Power Sybr Green Master Mix (Life Technologies) on a VIIA7 (Life Technologies) machine. Mean values were normalized to *RPS16* and fold change was calculated using the indicated controls. Experiments were carried out in biological triplicates (with the exception of PHH and fetal liver which were only measures once) and are depicted as mean values with 95% confidence interval (CI). Unpaired Student's *t*-tests were performed for calculating significances in Fig. 1, in all other cases ANOVA was used followed by Tukey’s multiple comparisons of means with 95% family wise confidence levels.

### Transcriptome and bioinformatics analysis

Microarray experiments were performed on human Clariom S Arrays (Affymetrix) (BMFZ, Düsseldorf).

### Data analysis

Untreated control HLCs and HLCs treated with AdipoRon, OA, and OA plus AdipoRon were hybridized on the Affymetrix Human Clariom S platform where CEL files were generated. These CEL files – regarded as the Affymetrix raw data – were read into the R/Bioconductor statistical package (Gentleman et al., 2004). The R package oligo was employed for background-correction and normalization via the Robust Multi-array Average (RMA) method (Carvalho and Irizarry, 2010). A detection *P*-value was calculated according to the method described in our previous publication by Graffmann et al. (Graffmann et al., 2016). A detection *P*-value of less than 0.05 was used to determine gene expression. Venn diagrams of expressed genes were made via the method venn from the gplots R package (Warnes et al., 2015), the dendrogram via the R function hclust. In order to determine differentially expressed genes the Bioconductor packages limma (Smyth, 2004) and qvalue (Storey, 2002) were applied.

### GO and pathway analysis

Over-represented GOs were assessed with the R package GOstats (Falcon and Gentleman, 2007). For determination of over-represented KEGG pathways (Kanehisa et al., 2017) a download of pathways and associated gene symbols from March 2018 was used (Fig. 5D,E). Over-representation was calculated with the R-built-in hypergeometric test. Dot plots of most significant terms were generated via the ggplot package (Wickham, 2009). Alternatively, up-and down-regulated genes were analysed with DAVID to derive KEGG-pathways (Fig. 5F-I) (Huang et al., 2009a,b). Metascape was used to analyse the commonly up-regulated GOs and Pathways of AdipoRon treated control and OA cells (Zhou et al., 2019).

## Supplementary Material

Supplementary information
