# Supplementary material for: A stem cell based in vitro model of NAFLD enables the analysis of patient specific individual metabolic adaptations in response to a high fat diet and AdipoRon interference
Source: Biol Open. 2021 Jan 25;10(1):bio054189. doi: 10.1242/bio.054189 (PMC7860118; doi:10.1242/bio.054189)
Supplement: Supplementary information [file biolopen-10-054189-s1.pdf]

Supplementary Fig. S1

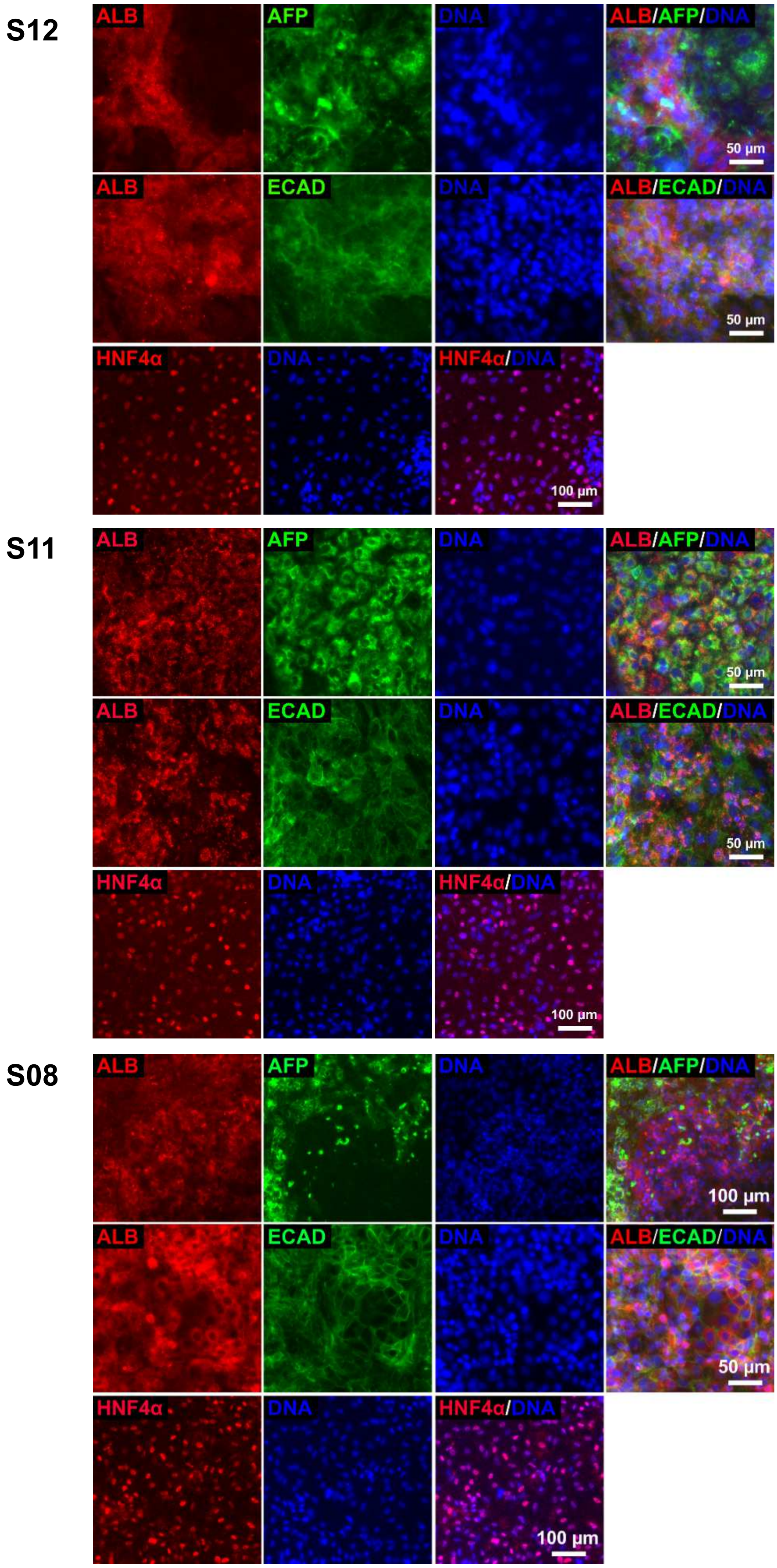

Fig. S1: Characterization of HLCs. Representative immunocytochemistry of hepatocyte markers at the end of HLC differentiation for the lines S12, S11, and S08. Cells were stained for ALB (red) and AFP (green) (upper lane), ALB (red) and ECAD (green) middle lane, HNF4α (red) (lower lane). DNA was stained with Hoechst 33258.

Supplementary Fig. S2

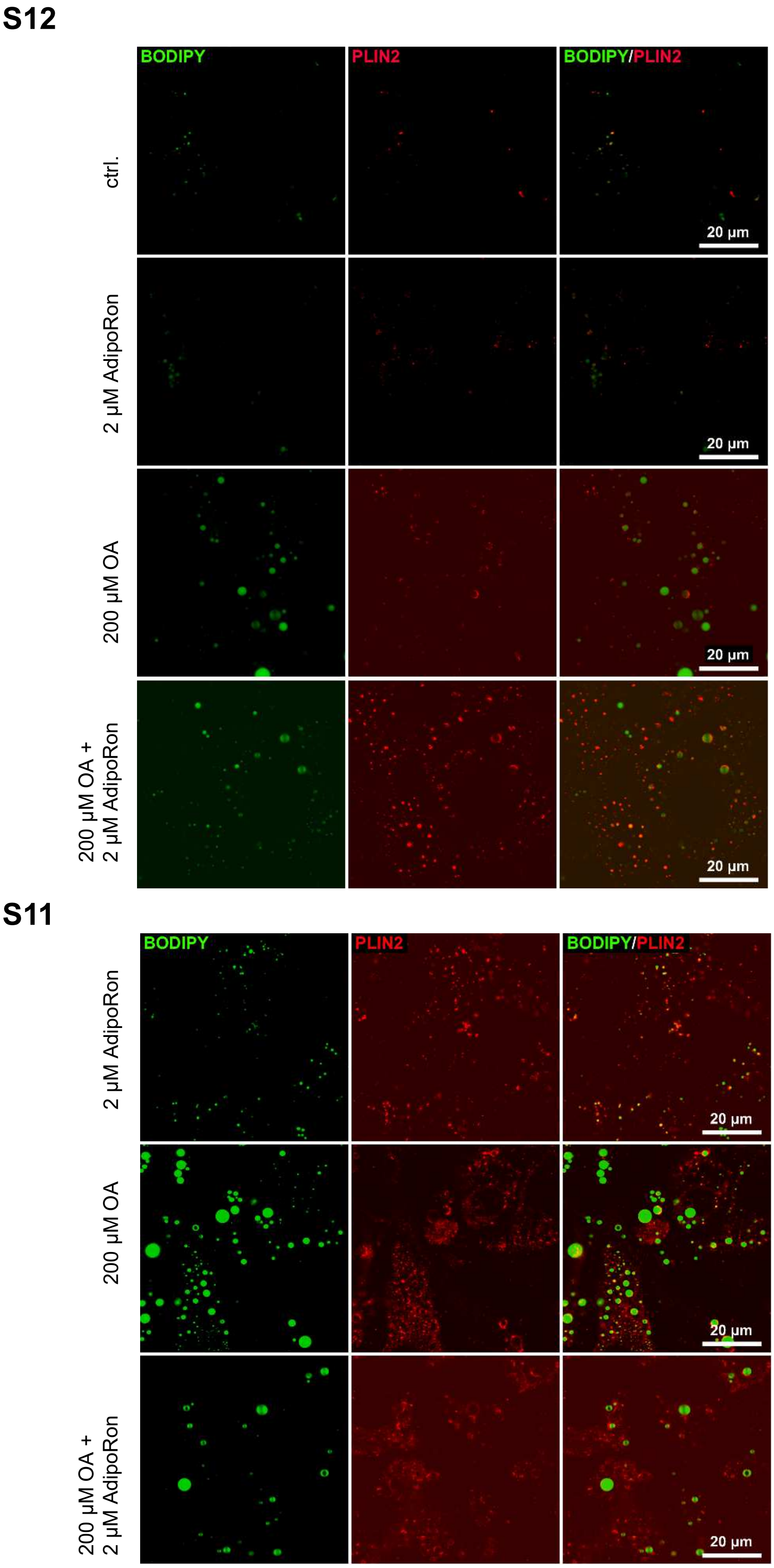

Fig. S2: Confocal pictures of HLCs. Representative immunocytochemistry of hepatocyte markers at the end of HLC differentiation for the lines S12 and S11. LDs (BODIPY 493/593, green), PLIN2 (red).

Biology Open • Supplementary information

Supplementary Fig. S3

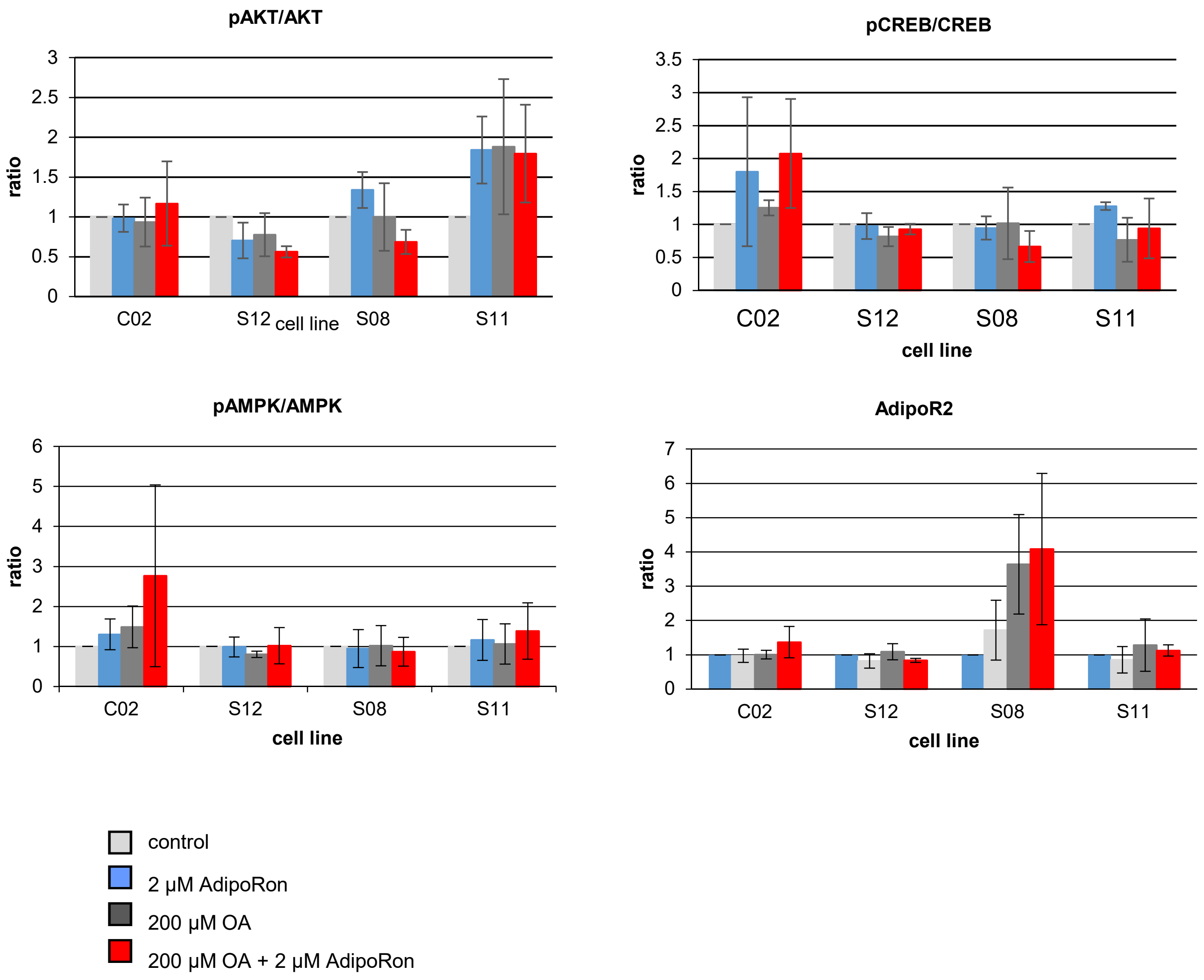

Fig. S3: Quantification of Western Blots for metabolic master regulators. Western Blots for the indicated factors as well as for β-ACTIN were performed in triplicates for each cell line. All bands were normalized to β-ACTIN. The ratio between the phosphorylated and the non-phosphorylated isoform was calculated. Depicted are means of 3 biological replicates +/- SD.

Biology Open • Supplementary information

Supplementary Fig. S4

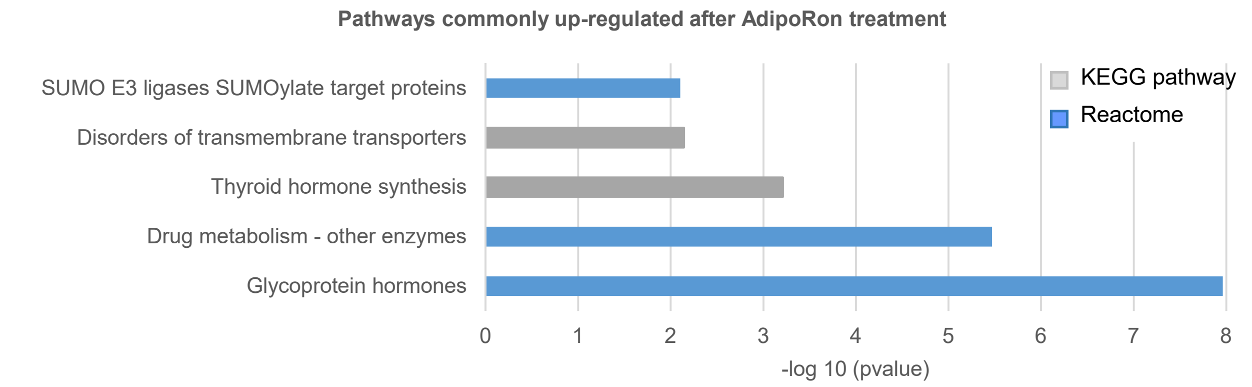

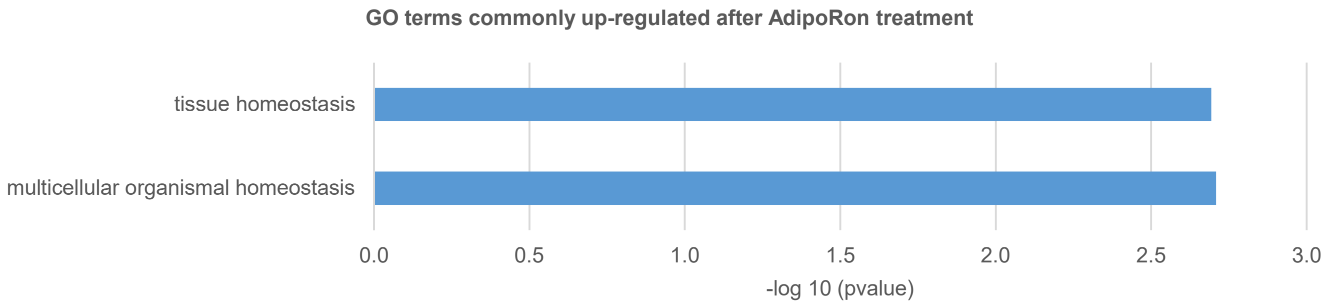

Fig. S4: Commonly AdipoRon regulated genes. Metascape was used to perform pathway enrichment analysis for genes that were commonly up- and down- regulated in control and OA treated cells.

Biology Open • Supplementary information

Table S1

Click here to Download Table S1

Table S2: Antibodies

Antibody	species	company	Order number	dilution	Application	Blocking
ALB	mouse	Sigma	A6684-.2ml	1:500	ICC	10% normal goat serum
AFP	rabbit	Sigma	HPA023600	1:250	ICC	10% normal goat serum
ECAD	rabbit	CST	3195	1:200	ICC	10% normal goat serum
HNF4α	rabbit	Abcam	92378	1:250	ICC	10% normal goat serum
PLIN2	rabbit	Proteintech	15294-1-AP	1:200	ICC	10% normal goat serum
AKT	rabbit	CST	9272S	1:1000	WB	5% milk TBST
pAKT	rabbit	CST	9271S	1:1000	WB	5% milk TBST
CREB	rabbit	CST	9197S	1:1000	WB	5% milk TBST
pCREB (Ser133)	rabbit	CST	9198S	1:1000	WB	5% milk TBST
AMPK	rabbit	CST	2532S	1:1000	WB	5% milk TBST
pAMPK (Thr172)	rabbit	CST	2535S	1:1000	WB	5% milk TBST
FGF21	rabbit	Abcam	Ab171941	1:1000	WB	5% milk TBST
AdipoR1	mouse	Proteintech	66619-1-Ig	1:1000	WB	5% milk TBST
AdipoR2	rabbit	Proteintech	14361-1-AP	1:500	WB	5% milk TBST
B-Actin	mouse	CST	3700S	1:5000	WB	5% milk TBST

Table S3

Click here to Download Table S3

Biology Open • Supplementary information
